# Human presence shifts the landscape of fear for a free‐living mammal

**DOI:** 10.1002/ecy.4499

**Published:** 2025-01-12

**Authors:** Chelsea A. Ortiz‐Jimenez, Sophie Z. Conroy, Erin S. Person, Jasper DeCuir, Gabriella E. C. Gall, Andrew Sih, Jennifer E. Smith

**Affiliations:** ^1^ Department of Environmental Science and Policy University of California Davis California USA; ^2^ Department of Biology Mills College at Northeastern University Oakland California USA; ^3^ Museum of Vertebrate Zoology, Department of Integrative Biology University of California Berkeley California USA; ^4^ Zukunftskolleg University of Konstanz Konstanz Germany; ^5^ Centre for the Advanced Study of Collective Behaviour University of Konstanz Konstanz Germany; ^6^ Biology Department University of Wisconsin Eau Claire Eau Claire Wisconsin USA

**Keywords:** California ground squirrel, giving‐up density, landscape of fear, predation risk, risk‐sensitivity, safety cues

## Abstract

Humans may play a key role in providing small prey mammals spatial and temporal refuge from predators, but few studies have captured the heterogeneity of these effects across space and time. Global COVID‐19 lockdown restrictions offered a unique opportunity to investigate how a sudden change in human presence in a semi‐urban park impacted wildlife. Here, we quantify how changes in the spatial distributions of humans and natural predators influenced the landscape of fear for the California ground squirrel (*Otospermophilus beecheyi*) in a COVID‐19 pandemic (2020) and non‐COVID (2019) year. We used a structural equation modeling approach to explore the direct and indirect effects of human presence, predator presence, and habitat features on foraging that reflected fear responses (e.g., giving‐up densities [GUDs], number of foragers, and average food intake rate while at food patches). In 2019, humans and dogs had moderate effects on GUDs; squirrels were less fearful (lower GUDs) in areas frequently visited by humans and dogs, but the effects of raptors were weak. In contrast, in 2020, the effects of humans and dogs on GUDs were weak; squirrels were more fearful of high raptor activity, open sky, and ground cover. In both years, squirrels farthest from refuge were the most risk‐averse. Overall, our analyses revealed an increase in perceived risk from natural predators in 2020 associated with a change in the concentration of human presence. Thus, risk‐sensitive foraging was dynamic across space and time, depending on a complex interplay among human and dog activity, natural predators, and microhabitat features. Our findings elucidate the myriad ways humans directly and indirectly influence animal perception of safety and danger.

## INTRODUCTION

Animals live in environments with spatial and temporal variation in predation risk where the ability to locate areas of minimal risk is a key fitness determinant (Lima & Dill, 1990; Sih, 1987; Smith et al., 2019; Wirsing et al., 2021). One major advance in understanding how risk perception varies across space involves mapping prey responses across a landscape, broadly referred to as the “landscape of fear” (Gaynor et al., 2019; Iribarren & Kotler, 2012; Laundré et al., 2001, 2014). This powerful approach links predation risk and other features of the physical landscape to explain spatial variation in prey numbers and risk‐sensitive behaviors (e.g., time and energy spent foraging). Although there is mixed empirical evidence for spatial gradients of fear across landscapes (e.g., Fortin et al., 2005; Hammerschlag et al., 2015; Kohl et al., 2018), the “dynamic” landscape of fear framework was recently proposed to explicitly incorporate how changes over time may account for predictable spatiotemporal variation in risk perception (Palmer et al., 2022).

Given the expansion of human activities, there is growing interest in understanding landscapes of fear in human‐dominated habitats (Fardell et al., 2021; Lodberg‐Holm et al., 2019; Stillfried et al., 2017), yet it is poorly understood whether these effects are consistent over time. In many parks, the recent COVID‐19 pandemic provided a natural experiment for elucidating how human activity affects risk‐sensitive behaviors (Rutz et al., 2020). Human activity was substantially reduced in some parks but varied at those that remained open. These changes may have cascading effects on wildlife behavior (Short et al., 2024). Moreover, human presence has been shown to induce fear responses in carnivores (Smith et al., 2017), which in some cases, even exceed responses to natural predators (Ciuti et al., 2012). In contrast, even if prey fear humans, they can exploit spatial and temporal refuge created by predators avoiding humans (e.g., the “human shield effect;” Berger, 2007). For example, some species alter activity to coincide with times of increased human presence and reduced predator activity (Lamichhane et al., 2023; Suraci et al., 2019). It is striking, however, that very few studies have tracked shifts in these responses over time.

Small mammals experience considerable temporal and spatial variability in predation risk, and these risks are reflected by foraging decisions (Fairbanks & Dobson, 2007; Jacob & Brown, 2000; Kotler et al., 1991; Orrock et al., 2004). A useful tool for assessing spatial variation in perceived risk involves measuring the relative exploitation of depletable food patches. This approach yields information on spatial variation in giving‐up densities (GUDs; i.e., amount of food remaining in a patch), an inverse estimate of foraging intensity. A higher GUD at a given location is commonly viewed as an indicator of higher risk. By mapping surrounding environmental factors (e.g., spatial variation in predator activity or habitat features), we can assess how factors affect foraging. GUDs thus offer an integrated measure of habitat‐specific risk perception (Bedoya‐Perez et al., 2013).

Although many studies have used GUDs to assess population‐level foraging decisions across a landscape of fear (Juliana et al., 2017; Kotler, 1997; Menezes et al., 2019; Toscano et al., 2016), these often represent the end result of multiple visitors foraging at each patch for varying amounts of time. Yet, the extent to which relative safety at patches adheres to a widely cited framework for understanding three hierarchical scales of habitat selection remains understudied (Johnson, 1980). First‐order decisions involve individual selection of home ranges that include a given patch. Areas where individuals perceive lower risk are likely to be visited more frequently and thus have higher local densities. Second‐order decisions pertain to microhabitat use in home ranges. Even if an individual deems an area “safe to visit” that does not necessarily mean that individuals will consider a particular patch “safe to forage,” especially if foraging patches are farther from refuge. Thus, measuring the number of unique individuals to visit and actively forage at a food patch can provide finer‐scale insights above and beyond the effects of local population density. Third‐order decisions involve the animal's use of the patches they visit. Food patches where individuals actively forage for longer per visit may reflect relatively low perceived risk. Safety in a food patch visited by multiple individuals (e.g., because local density is high) but for a short duration per visit differs from that in a food patch visited by fewer individuals for longer. Finally, animals can vary their food intake rate while visiting a patch. If predation risk increases vigilance, this can reduce food intake rates at a patch. Conversely, risk might induce prey to feed faster to reduce their duration of risky exposure at the foraging patch.

We explored the landscape of fear at these different scales for the California ground squirrel (*Otospermophilus beecheyi*) in a semi‐urban park with varying rates of human and predator presence and diverse microhabitat features. Here we refer to the ecological concept of fear as measured by behavioral avoidance of perceived risks, rather than an emotional or physiological response (Ortiz‐Jimenez et al., 2022; Zanette & Clinchy, 2019); we previously linked behavioral and physiological fear responses in this population (Hammond et al., 2019).

We quantified fear across the landscape by variation in (1) GUDs, (2) local forager density, (3) number of unique foragers visiting a patch, (4) average time foragers spent per visit, and (5) food intake rate during the visit. We estimated the direct and indirect effects of human and dog activity, natural predators, and key habitat features on variation in prey behavior at multiple scales. We expected humans to provide refuge, yielding a negative correlation between humans and natural predators. We also expected a weaker effect of humans on spatial variation in GUDs and a concomitantly stronger effect of natural predators and habitat features that affect risk from natural predators when human visits were reduced. Finally, we expected increased perceived risk in areas far from refuge (burrows; Ortiz‐Jimenez et al., 2022), with low vegetation (more risk from ground predators; Ortiz et al., 2019), and low sky cover (exposed to raptors).

## METHODS

### Study site and subjects

We studied free‐living California ground squirrels at Briones Regional Park in Contra Costa County, California, USA (latitude: 37.93 N, longitude: 122.13 W, elevation: 319 m above mean sea level). This facultatively social species (Person et al., 2024) spends most of the day aboveground (Smith et al., 2018). It seeks safety in burrows to escape predators (e.g., rattlesnakes [*Crotalus oreganus*], coyotes [*Canis latrans*], red‐tailed hawks [*Buteo jamaicensis*], Cooper's hawks [*Accipiter cooperii*], white‐tailed kites [*Elanus leucurus*; Linsdale, 1946; Owings et al., 1977]). It mainly consumes seeds and plants (Augustine et al., 2023; Evans & Holdenried, 1943; Fitch, 1948; Smith et al., 2016), but also opportunistically hunts vertebrate prey (Smith et al., Forthcoming). Food abundance influences its movement decisions (Dobson, 1979).

The study site was an old walnut grove (~0.96 ha; Ortiz et al., 2019) near a popular hiking trail and part of ongoing (since 2013) ecological monitoring. Foot traffic by humans and (on and off‐leash) domestic dogs varied over the site, according to paths and human amenities (Hammond et al., 2019). The current study was conducted in 2019 (year before the global COVID‐19 pandemic) and 2020 (first year of the pandemic). Monitoring was from late May to early August, annual periods of increased squirrel activity (Tomich, 1962). On a biweekly schedule, we trapped squirrels using Tomahawk Live Traps (Hazlehurst, Wisconsin, USA) baited with black oil sunflower seeds and peanut butter (Person et al., 2023). Each individual was marked with a uniquely numbered Monel ear tag (National Brand and Tag Co., Newport, Kentucky, USA) and a Passive Integrated Transponder (PIT) tag (Biomark Inc., Idaho, Nebraska, USA). For visual identification, each individual also received a unique fur mark (Nyanzol Dye, Greenville Colorants, Jersey City, New Jersey, USA). After processing, ground squirrels were released where captured. All procedures were approved by the IACUC Committees at Mills College and the University of California at Davis IACUC protocol #19853 and consistent with the guidelines of the American Society of Mammologists for the use of wild mammals in research (Sikes, 2016).

### Spatial observations

Space use was observed from May 24 to July 31 in 2019 (198 h) and June 12 to August 4 in 2020 (116 h) on weekdays from 0800 to 1200 h. We recorded marked squirrels that approached ≤5 m from natural and artificial landmarks (e.g., trees, logs, picnic benches, outhouse) (Gall et al., 2022). We noted the location of diurnal rattlesnakes, raptors, humans, and dogs. Despite being an important determinant of risk‐sensitive behavior (Smith et al., 2023), coyotes at our site are mainly nocturnal (unpublished camera trap data), and their diurnal activity was excluded from the current study because it was only noted during 10 and 3 observation days, respectively, in 2019 and 2020. To quantify the distribution and activity of diurnal visitors, we divided the number of individuals of each visitor/predator type (e.g., rattlesnakes, raptors) present ≤15 m from each landmark (Gall et al., 2022) by the number of observation hours that day. We averaged all observation days from a season to yield seasonal activity scores for each species per location (Ortiz‐Jimenez et al., 2022). Predator distributions were reasonably stable within each summer, and thus we mapped the average relative presence of humans and predator types across the site for each year (Brown, 1999).

### Giving‐up densities

To investigate habitat preference and perceived risk, GUD trials (estimates of foraging intensity) were run three times each year, roughly every 2 weeks between late June and end of July. We placed depletable food patches across the site in a 10 × 10 m grid (Appendix S1: Figure S1). Food patches consisted of a transparent plastic plate (36 cm diameter; Vigoro, Chicago, Illinois, USA) filled with 2 L of Cemex 30 mesh playbox sand and 5 g of millet (*Pennisetum glaucum*; a low‐quality but avidly consumed food source by squirrels; Brown, 1988). Each summer, roughly 100 plates were deployed in a permanent grid (2019 = 98 plates, 2020 = 106 plates); in 2020, we added a few plates to cover edges of the study site (Appendix S1: Figure S1). Millet was evenly mixed throughout sand and a few black oil sunflower seeds were placed on top as an initial lure. Plates were set out from 0800 to 1200 h. Observers (Appendix S1: Figure S1) silently noted the identity and duration of squirrels foraging at plates. Because nontarget visitors could contribute to GUDs (Gaynor et al., 2019), we noted if songbirds (e.g., oak titmouse [*Baeolophus inornatus*], scrub jay [*Aphelocoma californica*], American crow [*Corvus brachyrhynchos*]), or fox squirrels (*Sciurus niger*) visited plates and included this covariate in the analysis (hereafter “nontarget forager”). At the end of each trial, the remaining millet was recovered from the sand using a stainless‐steel mesh sifting pan and weighed using an Ohaus scale (±0.001 g). We focused on three outcomes: (1) proportion of millet remaining (GUD), (2) number of unique foragers who visited the plates, and (3) average time a forager spent on each plate during each four‐hour trial.

### Squirrel density

For each trial, we used the spatial data of all squirrels combined across a 2‐week period prior to the trial and calculated the utilization distribution (UD) using a kernel density estimation (grid size = 5 m). We then mapped the plate locations onto the UDs and calculated the local squirrel density for each plate as the UD of the nearest point on the UDs grid.

### Microhabitat

We assessed microhabitat features by taking a series of photographs at each plate used in GUD trials. To generate a vegetation index, we constructed a quadrat (83.5 cm × 98.0 cm) out of PVC pipes and scored each of the 36 (6 × 6) squares (dirt = 0, leaf litter = 0.5, living vegetation = 1) to generate a composite numerical score (hereafter “ground cover”). In addition, photos of tree cover were taken from the ground; a grid of 36 squares was overlaid on photos to assign the proportion of tree coverage (open sky = 0, tree vegetation = 1; hereafter “sky cover”). Lastly, we measured the distance to nearest burrow from each plate (Ortiz‐Jimenez et al., 2022).

### Structural equation modeling conceptual framework

**FIGURE 1:**
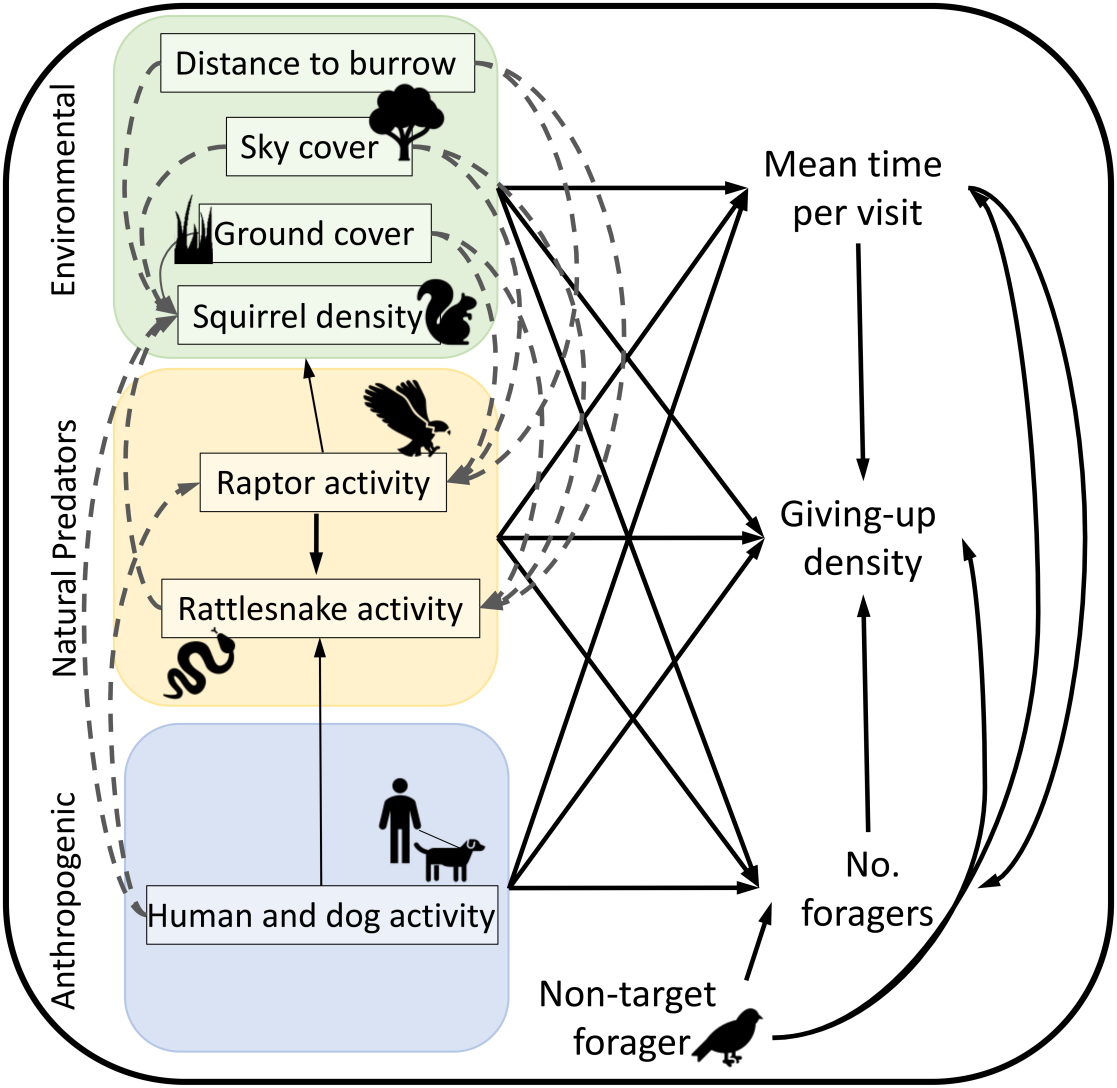
A priori model of all variables included in the model and all biologically relevant paths. Dotted paths represent indirect relationships between predictor variables, solid lines represent direct relationships between predictor variables and outcome variables.

To analyze the direct and indirect relationships of multiple predictor variables and our three main outcome variables, we implemented a structural equation modeling (SEM) approach (see Figure 1). SEMs are a widely recognized tool for exploring causal relationships among ecological variables (Garrido et al., 2022; Shipley, 2009). A major goal of the current study was to understand variation in GUDs (amount of food remaining), where less food remaining reflects less caution during foraging. GUDs depend on the number of foragers and mean time per visit and on relative individual feeding rates. Although we did not measure feeding rates directly, we can estimate seed consumption based on estimates of time spent visiting plates and seeds remaining (e.g., seeds removed and presumably consumed) for GUDs. In the SEM, we predicted that higher local squirrel density should reduce GUDs at a given patch by increasing the number of foragers visiting that patch, and if higher local density makes squirrels safer, that could result in longer mean times per visit and higher feeding rates (seeds eaten per second) during visits (Figure 1). The direct effect on GUDs is captured by the arrow going directly from a given factor to the GUD, while indirect effects can flow through several multi‐arrow, indirect pathways. For example, if squirrels fear humans and dogs, then humans and dogs can affect squirrel GUDs indirectly by reducing local squirrel density, number of unique squirrel visitors, and time per visit and directly by influencing intake rates by squirrels while visiting a given patch. Humans could also indirectly affect squirrel GUDs, by influencing the spatial pattern of rattlesnake or raptor activity that, in turn, can affect local squirrel density, the number of squirrels visiting a patch, average time per visit, and intake rates while in the patch. Habitat characteristics such as distance to refuge and habitat structure (e.g., ground cover and sky cover), predator activity, and the activity of humans (and dogs) may each affect GUDs via multiple direct and indirect pathways.

### Statistical implementation of SEMs

Because of substantial differences, we conservatively analyzed data from 2019 to 2020 separately. We implemented piecewise SEMs using the package piecewiseSEM (Lefcheck, 2016) to investigate direct and indirect relationships among factors (Santillán et al., 2020). Before constructing SEMs, we investigated correlations between variables. Because we uncovered high correlations between human activity and dog activity for both years (2019: *r* = 0.85; 2020: *r* = 0.98), we combined them into one composite variable (hereafter as “human and dog activity”). We tested the strength of the correlation between our remaining independent variables; all variance inflation factors (VIFs) showed no or low correlation values. For residual correlations greater than 0.10, we told the model the direction of the arrow based on our a priori biological understanding of the relationships between the factors (Lefcheck, 2016).

We constructed a fully saturated a priori model (Figure 1), which explored the causal relationship between GUDs, numbers of unique foragers, average time a forager spent at a plate (henceforth “mean time per visit”), natural predator activity, microhabitat variables, anthropogenic variables, and our control for nontarget foragers (recorded as a binary [0/1] response if a nontarget forager went to the plate during a trial). This saturated model included the maximum number of biologically plausible relationships (henceforth “paths”) between variables (Antiqueira et al., 2020; Garrido et al., 2022) specified by lmer() and glmer() functions from the R package *lme4* (Bates et al., 2015) with “plate ID” included as a random effect. All variables were standardized. After testing for normality of model residuals, we applied the arcsine transformation to two variables (proportion of millet remaining and local squirrel density) and square root transformations to rattlesnake and raptor variables to meet assumptions of normality.

We used Shipley's test of d‐separation to identify nonsignificant paths and removed paths that lowered the Bayesian information criterion (BIC) of each SEM. We selected final models based on the lowest BIC (Garrido et al., 2022; Lin et al., 2017) and goodness‐of‐fit test (Appendix S1: Tables S1 and S2). Goodness‐of‐fit was determined by Fisher's C test, with model acceptance when *p* > 0.05 (Shipley, 2009). We then calculated the direct, indirect, and net effects of variables, using the package semEFF (Murphy, 2022; Table 1; Appendix S1: Tables S3 and S4). Effects sizes were adjusted for multicollinearity among predictors (Petraitis et al., 1996) and standardized to zero mean and unit variation; we report semi‐partial correlations bounded between −1 and 1 (Murphy, 2022). Data processing and analyses were conducted in R version 4.1.1 (R Core Team, 2021). We assumed significance at the α≤0.05 level.

**TABLE 1:** Standardized direct, indirect, and net estimates of predictor variables on the giving‐up density of ground squirrels in 2019 and 2020.

Predictor variables	2019—Standard estimates			2020—Standard estimates		
	Direct effects	Indirect effects	Net effects	Direct effects	Indirect effects	Net effects
Mean time per visit	**−0.091** (−0.170, −0.014)	…	**−0.091** (−0.170, −0.014)	**−0.260** (−0.344, −0.195)	…	**−0.260** (−0.344, −0.195)
No. unique foragers	**−0.103** (−0.202, −0.026)	**−0.068** (−0.126, 0.013)	**−0.171** (−0.258, −0.105)	**−0.318** (−0.413, −0.289)	**−0.166** (−0.215, −0.117)	**−0.484** (−0.567, −0.465)
Squirrel density	**−0.289** (−0.371, −0.182)	**−0.061** (−0.099, −0.029)	**−0.350** (−0.421, −0.246)	…	**−0.092** (−0.134, −0.025)	**−0.092** (−0.134, −0.025)
Human and dog activity	**−0.254** (−0.358, −0.161)	**0.087** (0.018, 0.155)	**−0.167** (−0.26, −0.08)	…	**0.092** (0.055, 0.183)	**0.092** (0.055, 0.183)
Rattlesnake activity	…	**−0.096** (−0.153, −0.049)	**−0.096** (−0.153, −0.049)	…	**−0.018** (−0.037, −0.001)	**−0.018** (−0.037, −0.001)
Raptor activity	…	**−0.012** (−0.039, 0.006)	**−0.012** (−0.039, 0.006)	…	**0.105** (0.073, 0.162)	**0.105** (0.073, 0.162)
Distance to burrow	**0.164** (0.047, 0.270)	**0.163** (0.12, 0.217)	**0.327** (0.212, 0.435)	…	**0.148** (0.119, 0.218)	**0.148** (0.119, 0.218)
Ground cover	…	**0.069** (0.037, 0.120)	**0.069** (0.037, 0.12)	**0.164** (0.072, 0.212)	**0.093** (0.059, 0.152)	**0.257** (0.148, 0.345)
Sky cover	…	**−0.013** (−0.068, 0.048)	**−0.013** (−0.068, 0.048)	**−0.139** (−0.218, −0.072)	**−0.031** (−0.056, −0.017)	**−0.170** (−0.257, −0.100)
Nontarget forager	…	…	…	…	…	…

*Note*: CIs were calculated using 5000 bootstrapping resamples (95% CIs). Ellipsis reflects variables not retained in model. Bolded values were statistically signifcant at an alpha of less than 0.05.

## RESULTS

Overall, we observed a total of 241 individual squirrels during GUD trials. Of these individuals, 106 squirrels (43%) visited plates in both years. The mean ± SE amount of millet at the end of each trial was similar in 2019 (2.31 ± 0.10 g, range: 0.004–5.06 g) and 2020 (2.20 ± 0.10 g, range: 0.11–4.94 g). The number of unique foragers at each plate in 2019 (2.3 ± 0.17 foragers, range: 0–19 foragers) was also comparable to 2020 (2.1 ± 0.11 foragers, range: 0–9 foragers). However, squirrels foraged, on average, for 1.5 times longer on plates in 2020 (2.4 ± 0.15 min, range: 0–22 min) than in 2019 (1.6 ± 0.10 min, range: 0–10 min).

### Spatial distribution of humans, predators, and squirrel activity

**FIGURE 2:**
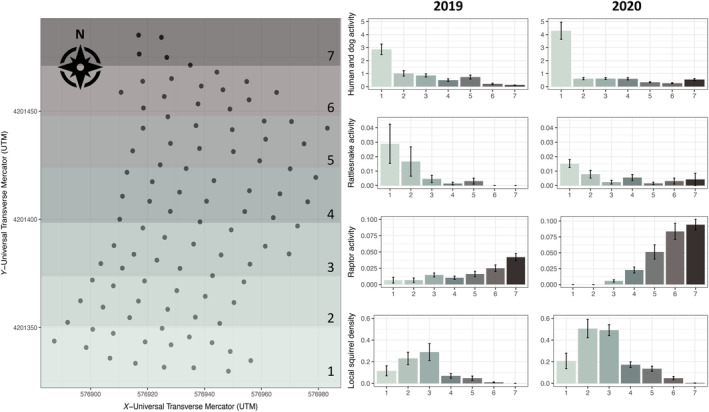
(a) Spatial map of study site with plate locations (circles) separated into seven geographic regions from north (black) to south (light green) and (b) average activity from all visitor types (humans and dogs, rattlesnakes, and raptors) and average local squirrel density from plates in the associated regions from the spatial map. Error bars indicate SEs.

The spatial distribution and variation in visits by humans and dogs and raptors differed between years (Figure 2b). Whereas the overall hourly rate of visits to the study site was not markedly different between years for humans and dogs or rattlesnakes (Appendix S1: Tables S5 and S6; Figure 2), raptor visits were twice as common in 2020 than in 2019. In addition, the spatial concentration of visitors differed between years. In 2020, human and dog activity was concentrated more in the southernmost region of the study area but was more evenly distributed over the site in 2019. Specifically, humans and dogs visited regions two, three, and five less in 2020 than in 2019, but raptors visited regions 4–7 more in 2020 than in 2019; rattlesnake activity was more evenly distributed across regions in 2020 than in 2019 (Figure 2). Roughly 1.5 times more squirrels visited per plate in 2020 than in 2019 (0.48 ± 0.20 vs. 0.33 ± 0.20, Figure 2b). From 2019 to 2020, local squirrel density doubled overall, increasing mostly in regions 2–6.

### Effects of squirrel density, and visits to trays on GUDs

**FIGURE 3:**
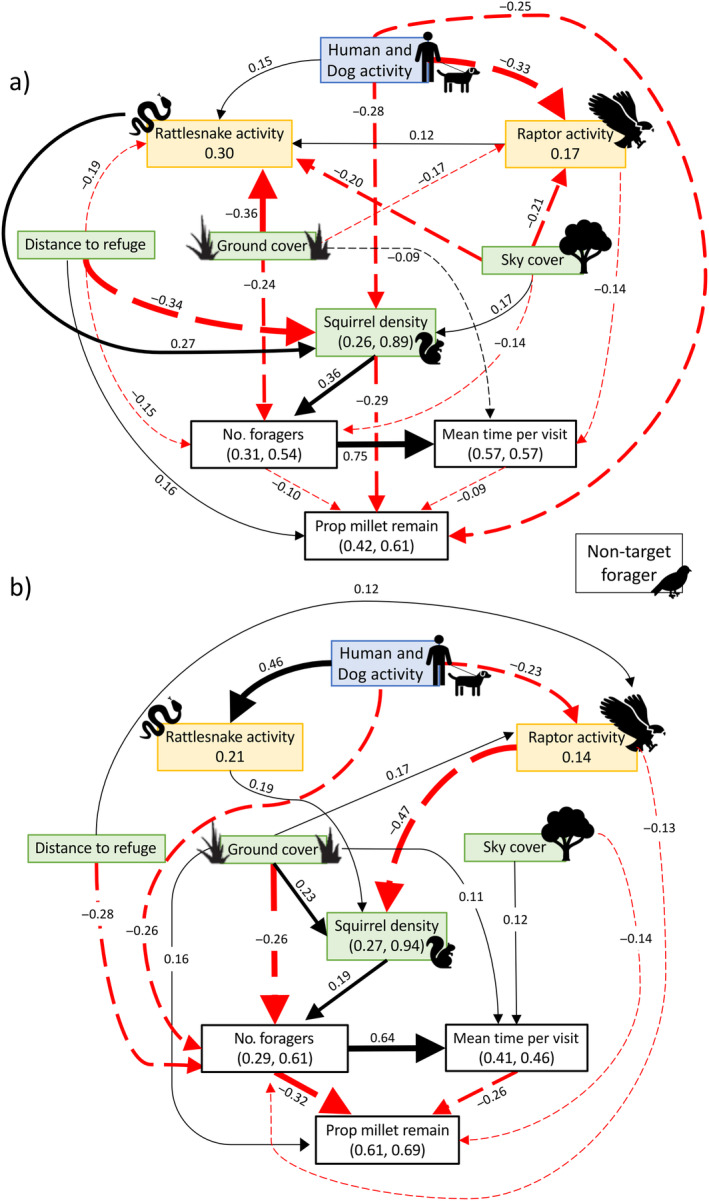
Final piecewise structural equation model (SEM) for (a) 2019 and (b) 2020. Each variable is shown in a box, with dependent variables shown with the marginal *R* ^2^ and conditional *R* ^2^ values. For both SEMs, arrows represent unidirectional relationships among the variables with solid lines representing positive effects and dashed lines representing negative effects. The number along each path (arrows) is the standardized estimate while the line thickness represents the strength with thicker lines having a stronger effect.

With regard to the factors affecting GUDs, less food remained in plates when they were visited by more squirrels that stayed longer (Figure 3, Table 1). The net effects (and their effect sizes reported as associated partial correlations) were stronger in 2020 than 2019 (the number of unique foragers: −0.484 [−0.567, −0.465] vs. −0.170 [−0.258, −0.105]; mean time per visit: −0.260 [−0.344, −0.195] vs. −0.091 [−0.170, −0.014]; Table 1). In both years, patches in areas with high local squirrel density were visited by more unique foragers than patches with low local squirrel density. In 2019, but not in 2020, squirrels spent more time on plates and consumed seeds at a higher rate when visiting areas of high local squirrel density (Figure 3a,b, Table 1).

### Human, predator, and habitat effects on GUDs

In 2019, human and dog activity directly affected GUDs (i.e., more seeds were consumed from plates in areas with more human and dog activity; e.g., region 1 of Figure 2). Although human and dog activity was associated with lower local squirrel density (i.e., squirrels avoided areas of higher human and dog activity: ‐0.28 [‐0.456, ‐0.111]), human and dog activity had an overall negative effect on GUDs (Table 1; ‐0.167 (‐0.260,‐0.080). Rattlesnake activity was positively correlated with human and dog activity (Figure 3a) and also indirectly negatively correlated with GUDs: −0.096 (−0.153, −0.0.049). Raptor activity influenced mean time per visit where squirrels spent less time foraging on plates with high raptor presence; however, the overall effect on GUDs was weak. Distance to burrow had the strongest effect on GUDs: 0.327 (0.212, 0.435). Plates farther from burrow refuge had more food remaining (i.e., fear increased with distance from refuge) because they received fewer visits, with shorter average time per visit, and lower food intake rate while at plates. Increased sky cover and ground cover were also associated with more food remaining in plates, but these effects were relatively weak.

In 2020, human and dog activity had weaker effects on GUDs and other variables than in 2019. Human and dog activity failed to significantly influence GUDs directly; indirect effects on GUDs were also weak: 0.092 (0.055, 0.183). The effects of rattlesnake activity, human and dog activity, and local squirrel density on lowering GUDs (i.e., less food remaining) were weak. In contrast, the weak indirect effect of raptor activity on GUDs increased in 2020: 0.105 (0.073, 0.162), compared with 2019. Squirrels actively avoided areas with higher raptor activity (i.e., lower local squirrel density) and had shorter visits to areas where raptor activity was higher, which resulted in more food left in plates. We also found a negative relationship between raptor activity and human and dog activity (e.g., fewer raptors in areas of high human and dog activity). Raptors avoided the high human and dog areas (e.g., plates in region 1 of Figure 2) while squirrels foraged less in the areas where raptors resided (e.g., plates in regions 5–7 of Figure 2). With the increase in raptor presence on the northern portion of the site in 2020, microhabitat features played a larger role than in 2019. Squirrels left more food in areas with more ground cover, with a moderate net effect: 0.257 (0.148, 0.345); high ground cover had a weak positive direct (0.164 [0.072, 0.212]) and indirect (0.093 [0.059, −0.017]) effects on GUDs, reducing the number of visitors and food intake rates during visits. In contrast, squirrels ate more food (−0.170 [−0.257, −0.100]) and spent longer foraging on plates with more sky cover, but sky cover did not predict the number of foragers (Appendix S1: Tables S3 and S4).

## DISCUSSION

Overall, we found that changes in human behavior and space use due to pandemic disruption influenced ground squirrel risk perception, providing support for the human shield hypothesis (Berger, 2007). In 2019, human and dog presence had moderate direct effects on the amount of food consumed (e.g., more seeds consumed in human‐influenced areas); net effects of humans on seed consumption were slightly reduced by their weak indirect effects on GUDs (Cohen, 1992). However, in 2020, human and dog presence only imposed small, indirect effects on GUDs (Cohen, 1992). Thus, as human, dog, and raptor presence shifted between years, different factors altered how squirrels navigated the landscape of fear at different scales. These ranged from avoidance of an area altogether to subtle changes in the numbers of foragers visiting a patch, time spent foraging at a patch, and seed consumption rate during visits. While GUDs alone did provide information about fearfulness across the landscape, our added measures of the number of unique foragers, average time spent foraging per visit, and feeding rate at a patch provided finer scale details from individually recognized squirrels.

Consistent with the “dynamic” landscape of fear framework (Palmer et al., 2022), we found that a shift in visitor space use between years had significant impacts on risk‐sensitive foraging. In 2019, the pre‐COVID year, when human and dog activity was less concentrated in one area, squirrels were less fearful (had lower GUDs) in areas of high human and dog activity. This fits the “enemy of my enemy is my friend” idea seen in other systems (Caldwell & Klip, 2022; Kuijper et al., 2015; Leighton et al., 2010; Suraci et al., 2019) where human presence can be beneficial for prey, perhaps by reducing predation risk from real predators. Importantly, however, our ability to partition direct and indirect pathways suggested that squirrels are not “fearless” of humans but rather exhibited temporal and spatial heterogeneities in risk perception associated with patterns of human and dog activity. In 2019, on a weekly scale, squirrels showed a tendency to avoid areas with higher human and dog activity, but when squirrels used those areas, their foraging intake rate was significantly higher (i.e., lower GUDs, less fear). In contrast, in 2020, we did not see any direct relationship between human and dog activity and foraging rate even though overall human and dog activity was similar to the previous year. Perhaps because human and dog activity was strongly concentrated in one region of the study site, it had only a small effect on squirrel GUDs. Squirrels tended to avoid areas with higher human and dog activity, to visit plates less often, and for shorter periods in 2020. These results suggest that beyond the effects of overall human activity per se, the spatial concentration of human activity can play a key role in how humans impact prey landscapes of fear.

For both years, raptor activity was negatively linked to human and dog activity; however, in 2020, the COVID year, when human and dog activity had little effect on squirrel GUDs, raptors had much stronger effects on squirrel GUDs than in 2019. We found that squirrels often avoided areas of higher raptor activity, but also expressed fear through shorter foraging times which significantly influenced GUDs. Overall, in the pre‐COVID year, the impact of raptors on GUDs was weak, while in the COVID year, the impact of raptors on GUDs (i.e., fear of raptors) was much stronger. This occurred even though the total overall activity of raptors did not differ between years but conceivably reflected the change in the spatial pattern of raptor activity associated with the more concentrated use of space by humans and dogs in 2020.

With the shift in human and dog space use, we also found a change in the relative extent to which microhabitat features predicted risk‐sensitive foraging by squirrels. In both years, squirrels' risk‐sensitivity strongly and consistently increased the farther they were from refuge, an expected finding for this species (Ortiz‐Jimenez et al., 2022). In the non‐COVID year, distance to refuge was the strongest microhabitat factor, affecting foraging rate both directly and indirectly. Distance to refuge also decreased local squirrel density which further decreased the number of foragers to visit a plate beyond squirrel's avoidance of these areas. In 2020, distance to refuge did not significantly affect local squirrel density but did indirectly affect the foraging rate through fewer foragers at plates and via shorter visit durations. More strikingly, with the change in the presence of raptors in the COVID year, squirrels altered their response to variation in ground and sky cover. Compared to the non‐COVID year, squirrels increased their foraging rate in areas with more sky cover from surrounding trees, suggesting they were less vigilant and more focused on foraging in these areas safe from raptors. Surprisingly, during the COVID year, while local squirrel density was higher in areas of increased ground cover (i.e., squirrels did not avoid areas of high vegetation and leaf litter), squirrels often chose not to forage in these areas in either year.

We found a positive relationship between local squirrel density and the activity of another natural predator, the rattlesnake. This finding is consistent at a larger scale, with species abundances for ground squirrels and rattlesnakes covarying across California (Poran et al., 1987). Rattlesnake activity was also positively associated with that of humans and dogs. We do not suggest that squirrels are choosing to be near rattlesnakes but rather that both squirrels and snakes may be using refuge provided by humans and dogs; raptors, including red‐tailed hawks, are active predators of ground squirrels as well as rattlesnakes (Fitch et al., 1946).

In conclusion, changes in human activity had significant impacts on how free‐living animals navigate and assess the landscape of fear. We contribute to growing evidence for how global shutdowns from the COVID‐19 epidemic affected wildlife (Zellmer et al., 2020) and support the “dynamic” landscape of fear framework (Palmer et al., 2022). These data support our a priori hypothesis about how human and predator distributions affect small prey. We captured shifts in fear responses to address long‐standing questions in urban ecology and extend our understanding of the effects of human presence on refuge use dynamics. Although animals have generally become increasingly nocturnal to avoid humans (Gaynor et al., 2018), COVID‐19 lockdowns reversed these trends for some species (Gordo et al., 2021). Globally, animal species responded quickly—and usually positively—to reductions in human presence (Bates et al., 2021). In particular, carnivore numbers and movements increased in urban areas during lockdowns (Silva‐Rodríguez et al., 2021). In contrast, we show *increased fear* among small prey mammals associated with *reductions in human activity* (i.e., “the anthropause”; Rutz et al., 2020). That is, although ground squirrels are not fearless of humans (e.g., shorter foraging bouts and avoidance of areas with high human activity), these prey animals were more fearful during lockdowns when they presumably lacked access to the spatial refuges from predators provided by humans. Importantly, however, our results suggest that how humans affect the squirrels' landscape of fear depends on a complex interplay between the spatial concentration of human (and dog) activity and responses of multiple predators mediated by several key landscape features. Thus, our findings offer new insights into the varied ways human presence—through direct and indirect effects within ecological communities—influences animal perception of safety and danger in a changing world.

## AUTHOR CONTRIBUTIONS

This experiment was carried out by Chelsea A. Ortiz‐Jimenez, Sophie Z. Conroy, Erin S. Person, Jasper DeCuir, and Jennifer E. Smith with additional help from Andrew Sih, Gabriella E. C. Gall, and other members of Team Squirrel who contributed to this long‐term study. Data were analyzed by Chelsea A. Ortiz‐Jimenez with support from Andrew Sih, Jennifer E. Smith, and Gabriella E. C. Gall. All authors contributed to revising the manuscript and gave final approval for its publication.

## CONFLICT OF INTEREST STATEMENT

The authors declare no conflicts of interest.

## Supporting information


Appendix S1.

## Data Availability

Data (Ortiz‐Jimenez et al., 2024a) are available in Dryad at https://doi.org/10.5061/dryad.pk0p2ngv4. Code (Ortiz‐Jimenez et al., 2024b) is available in Zenodo at https://doi.org/10.5281/zenodo.10535833.

## References

[ecy4499-bib-0001] Antiqueira, P. A. P. , P. M. de Omena , T. Gonçalves‐Souza , C. Vieira , G. H. Migliorini , M. F. Kersch‐Becker , T. N. Bernabé , F. C. Recalde , S. B. Gordillo , and G. Q. Romero . 2020. “Precipitation and Predation Risk Alter the Diversity and Behavior of Pollinators and Reduce Plant Fitness.” Oecologia 192: 745–753. 32016526 10.1007/s00442-020-04612-0

[ecy4499-bib-0002] Augustine, D. J. , J. E. Smith , A. D. Davidson , and P. Stapp . 2023. “Burrowing Rodents.” In Rangeland Wildlife Ecology and Conservation, edited by L. B. McNew, D. K., Hahlgren and J. L. Beck, 505–548. Cham: Springer International Publishing.

[ecy4499-bib-0003] Bates, A. E. , R. B. Primack , B. S. Biggar , T. J. Bird , M. E. Clinton , R. J. Command , C. Richards , et al. 2021. “Global COVID‐19 Lockdown Highlights Humans as both Threats and Custodians of the Environment.” Biological Conservation 263: 109175. 34035536 10.1016/j.biocon.2021.109175 PMC8135229

[ecy4499-bib-0004] Bates, D. , M. Mächler , B. M. Bolker , and S. C. Walker . 2015. “Fitting Linear Mixed‐Effects Models Using lme4.” Journal of Statistical Software 67(1): 1–48.

[ecy4499-bib-0005] Bedoya‐Perez, M. A. , A. J. R. Carthey , V. S. A. Mella , C. McArthur , and P. B. Banks . 2013. “A Practical Guide to Avoid Giving Up on Giving‐Up Densities.” Behavioral Ecology and Sociobiology 67: 1541–1553.

[ecy4499-bib-0006] Berger, J. 2007. “Fear, Human Shields and the Redistribution of Prey and Predators in Protected Areas.” Biology Letters 3: 620–623. 17925272 10.1098/rsbl.2007.0415 PMC2391231

[ecy4499-bib-0076] Brown, J. S. 1988. “Patch use as an Indicator of Habitat Preference, Predation Risk, and Competition.” Behavioral Ecology and Sociobiology 22: 37–47.

[ecy4499-bib-0007] Brown, J. S. 1999. “Vigilance, Patch Use and Habitat Selection: Foraging under Predation Risk.” Evolutionary Ecology Research 1: 49–71.

[ecy4499-bib-0008] Caldwell, M. R. , and J. M. K. Klip . 2022. “Patterns of Wildlife Activity and Predator‐Prey Dynamics in a Highly Touristed Area.” The Southwestern Naturalist 66: 35–47.

[ecy4499-bib-0009] Ciuti, S. , J. M. Northrup , T. B. Muhly , S. Simi , M. Musiani , J. A. Pitt , and M. S. Boyce . 2012. “Effects of Humans on Behaviour of Wildlife Exceed those of Natural Predators in a Landscape of Fear.” PLoS One 7: e50611. 23226330 10.1371/journal.pone.0050611 PMC3509092

[ecy4499-bib-0010] Cohen, J. 1992. “A Power Primer.” Psychological Bulletin 112: 155–159. 19565683 10.1037//0033-2909.112.1.155

[ecy4499-bib-0011] Dobson, F. S. 1979. “An Experimental Study of Dispersal in the California Ground Squirrel.” Ecology 60: 1103–1109.

[ecy4499-bib-0012] Evans, F. C. , and R. Holdenried . 1943. “A Population Study of the Beechey Ground Squirrel in Central California.” Journal of Mammalogy 24: 231.

[ecy4499-bib-0013] Fairbanks, B. , and F. S. Dobson . 2007. “Mechanisms of the Group‐Size Effect on Vigilance in Columbian Ground Squirrels: Dilution Versus Detection.” Animal Behaviour 73: 115–123.

[ecy4499-bib-0014] Fardell, L. L. , C. E. M. Nano , C. R. Pavey , and C. R. Dickman . 2021. “Small Prey Animal Habitat Use in Landscapes of Fear: Effects of Predator Presence and Human Activity along an Urban Disturbance Gradient.” Frontiers in Ecology and Evolution 9: 886.

[ecy4499-bib-0015] Fitch, B. H. S. , F. Swenson , and D. F. Tillotson . 1946. “Behavior and Food Habits of the Red‐Tailed Hawk.” The Condor 48: 205–237.

[ecy4499-bib-0016] Fitch, H. S. 1948. “Ecology of the California Ground Squirrel on Grazing Lands.” American Midland Naturalist 39: 513–596.

[ecy4499-bib-0017] Fortin, D. , H. L. Beyer , M. S. Boyce , D. W. Smith , T. Duchesne , and J. S. Mao . 2005. “Wolves Influence Elk Movements: Behavior Shapes a Trophic Cascade in Yellowstone National Park.” Ecology 86: 1320–1330.

[ecy4499-bib-0018] Gall, G. E. C. , J. C. Evans , M. J. Silk , C. A. Ortiz‐Jimenez , and J. E. Smith . 2022. “Short‐Term Social Dynamics Following Anthropogenic and Natural Disturbances in a Free‐Living Mammal.” Behavioral Ecology 33: 705–720.

[ecy4499-bib-0019] Garrido, M. , S. K. Hansen , R. Yaari , and H. Hawlena . 2022. “A Model Selection Approach to Structural Equation Modelling: A Critical Evaluation and a Road Map for Ecologists.” Methods in Ecology and Evolution 13: 42–53.

[ecy4499-bib-0020] Gaynor, K. M. , J. S. Brown , A. D. Middleton , M. E. Power , and J. S. Brashares . 2019. “Landscapes of Fear: Spatial Patterns of Risk Perception and Response.” Trends in Ecology & Evolution 34: 355–368. 30745252 10.1016/j.tree.2019.01.004

[ecy4499-bib-0021] Gaynor, K. M. , C. E. Hojnowski , N. H. Carter , and J. S. Brashares . 2018. “The Influence of Human Disturbance on Wildlife Nocturnality.” Science 360: 1232–1235. 29903973 10.1126/science.aar7121

[ecy4499-bib-0022] Gordo, O. , L. Brotons , S. Herrando , and G. Gargallo . 2021. “Rapid Behavioural Response of Urban Birds to COVID‐19 Lockdown.” Proceedings of the Royal Society B 288: 1–8. 10.1098/rspb.2020.2513 PMC7944088 33715437

[ecy4499-bib-0023] Hammerschlag, N. , A. C. Broderick , J. W. Coker , M. S. Coyne , M. Dodd , M. G. Frick , M. H. Godfrey , et al. 2015. “Evaluating the Landscape of Fear between Apex Predatory Sharks and Mobile Sea Turtles across a Large Dynamic Seascape.” Ecology 96: 2117–2126. 26405737 10.1890/14-2113.1

[ecy4499-bib-0024] Hammond, T. T. , M. Vo , C. T. Burton , L. L. Surber , E. A. Lacey , and J. E. Smith . 2019. “Physiological and Behavioral Responses to Anthropogenic Stressors in a Human‐Tolerant Mammal.” Journal of Mammalogy 100: 1928–1940.

[ecy4499-bib-0025] Iribarren, C. , and B. P. Kotler . 2012. “Foraging Patterns of Habitat Use Reveal Landscape of Fear of Nubian Ibex *Capra nubiana* .” Wildlife Biology 18: 194–201.

[ecy4499-bib-0026] Jacob, J. , and J. S. Brown . 2000. “Microhabitat Densities Use, Giving‐up Activity Behaviors in Common Anti‐Predator Behaviors in Common Voles.” Oikos 91: 131–138.

[ecy4499-bib-0027] Johnson, D. H. 1980. “The Comparison of Usage and Availability Measurements for Evaluating Resource Preference.” Ecology 61: 65–71.

[ecy4499-bib-0028] Juliana, J. R. S. , B. P. Kotler , N. Wielebnowski , and J. G. Cox . 2017. “Stress as an Adaptation I: Stress Hormones Are Correlated with Optimal Foraging Behaviour of Gerbils under the Risk of Predation.” Evolutionary Ecology Research 18: 571–585.

[ecy4499-bib-0029] Kohl, M. T. , D. R. Stahler , M. C. Metz , J. D. Forester , M. J. Kauffman , N. Varley , P. J. White , D. W. Smith , and D. R. MacNulty . 2018. “Diel Predator Activity Drives a Dynamic Landscape of Fear.” Ecological Monographs 88: 638–652.

[ecy4499-bib-0030] Kotler, B. P. 1997. “Patch Use by Gerbils in a Risky Environment: Manipulating Food and Safety to Test Four Models.” Oikos 78: 274–282.

[ecy4499-bib-0031] Kotler, B. P. , J. S. Brown , and O. Hasson . 1991. “Factors Affecting Gerbil Foraging Behavior and Rates of Owl Predation.” Ecology 72: 2249–2260.

[ecy4499-bib-0032] Kuijper, D. P. J. , J. W. Bubnicki , M. Churski , B. Mols , and P. Van Hooft . 2015. “Context Dependence of Risk Effects: Wolves and Tree Logs Create Patches of Fear in an Old‐Growth Forest.” Behavioral Ecology 26: 1558–1568.

[ecy4499-bib-0033] Lamichhane, S. , B. R. Lamichhane , A. Gurung , T. Rayamajhi , T. P. Dahal , P. R. Regmi , C. P. Pokheral , et al. 2023. “Non‐exploitative Human Disturbance Provides Shelter for Prey from Predator.” Ecology and Evolution 13: 1–10. 10.1002/ece3.10200 PMC10269119 37332517

[ecy4499-bib-0034] Laundré, J. W. , L. Hernández , and K. B. Altendorf . 2001. “Wolves, Elk, and Bison: Reestablishing the “Landscape of Fear” in Yellowstone National Park, U.S.A.” Canadian Journal of Zoology 79: 1401–1409.

[ecy4499-bib-0035] Laundré, J. W. , L. Hernández , P. L. Medina , A. Campanella , J. López‐Portillo , A. González‐Romero , K. M. Grajales‐Tam , A. M. Burke , P. Gronemeyer , and D. M. Browning . 2014. “The Landscape of Fear: The Missing Link to Understand Top‐Down and Bottom‐Up Controls of Prey Abundance?” Ecology 95: 1141–1152. 25000746 10.1890/13-1083.1

[ecy4499-bib-0036] Lefcheck, J. S. 2016. “piecewiseSEM: Piecewise Structural Equation Modelling in r for Ecology, Evolution, and Systematics.” Methods in Ecology and Evolution 7: 573–579.

[ecy4499-bib-0037] Leighton, P. A. , J. A. Horrocks , and D. L. Kramer . 2010. “Conservation and the Scarecrow Effect: Can Human Activity Benefit Threatened Species by Displacing Predators?” Biological Conservation 143: 2156–2163.

[ecy4499-bib-0038] Lima, S. L. , and L. M. Dill . 1990. “Behavioral Decisions Made under the Risk of Predation: A Review and Prospectus.” Canadian Journal of Zoology 68: 619–640.

[ecy4499-bib-0039] Lin, L. C. , P. H. Huang , and L. J. Weng . 2017. “Selecting Path Models in SEM: A Comparison of Model Selection Criteria.” Structural Equation Modeling 24: 855–869.

[ecy4499-bib-0040] Linsdale, J. M. 1946. The California Ground Squirrel: A Record of Observations Made on the Hastings Natural History Reservation. Berkeley, CA: University of California Press.

[ecy4499-bib-0041] Lodberg‐Holm, H. K. , H. W. Gelink , A. G. Hertel , J. E. Swenson , M. Domevscik , and S. M. J. G. Steyaert . 2019. “A Human‐Induced Landscape of Fear Influences Foraging Behavior of Brown Bears.” Basic and Applied Ecology 35: 18–27.

[ecy4499-bib-0042] Menezes, J. F. S. , B. P. Kotler , and A. K. Dixon . 2019. “Risk Pump in *Gerbillus pyramidum*: Quality of Poor Habitats Increases with More Conspecifics.” Ethology Ecology and Evolution 31: 140–154.

[ecy4499-bib-0043] Murphy, M. V. 2022. “Automatic Calculation of Effects for Piecewise Structural Equation Models.” R Package semEff. Comprehensive R Archive Network (CRAN).

[ecy4499-bib-0044] Orrock, J. L. , B. J. Danielson , and R. J. Brinkerhoff . 2004. “Rodent Foraging Is Affected by Indirect, but Not by Direct, Cues of Predation Risk.” Behavioral Ecology 15: 433–437.

[ecy4499-bib-0045] Ortiz, C. A. , E. L. Pendleton , K. L. Newcomb , and J. E. Smith . 2019. “Conspecific Presence and Microhabitat Features Influence Foraging Decisions across Ontogeny in a Facultatively Social Mammal.” Behavioral Ecology and Sociobiology 73: 42.

[ecy4499-bib-0046] Ortiz‐Jimenez, C. , S. Conroy , E. Person , J. DeCuir , G. Gall , A. Sih , and J. Smith . 2024a. “Human Presence Shifts the Landscape of Fear for a Free‐Living Mammal.” Dataset. Dryad. 10.5061/dryad.pk0p2ngv4. PMC11725700 39800902

[ecy4499-bib-0047] Ortiz‐Jimenez, C. , S. Conroy , E. Person , J. DeCuir , G. Gall , A. Sih , and J. Smith . 2024b. “Human Presence Shifts the Landscape of Fear for a Free‐Living Mammal.” Zenodo. 10.5281/zenodo.10535833. PMC11725700 39800902

[ecy4499-bib-0048] Ortiz‐Jimenez, C. A. , M. Michelangeli , E. Pendleton , A. Sih , and J. E. Smith . 2022. “Behavioural Correlations across Multiple Stages of the Antipredator Response: Do Animals that Escape Sooner Hide longer?” Animal Behaviour 185: 175–184.

[ecy4499-bib-0049] Owings, D. H. , M. Borchert , and R. Virginia . 1977. “The Behaviour of California Ground Squirrels.” Animal Behaviour 25: 221–230.

[ecy4499-bib-0050] Palmer, M. S. , K. M. Gaynor , J. A. Becker , J. O. Abraham , M. A. Mumma , and R. M. Pringle . 2022. “Dynamic Landscapes of Fear: Understanding Spatiotemporal Risk.” Trends in Ecology and Evolution 37: 911–925. 35817684 10.1016/j.tree.2022.06.007

[ecy4499-bib-0051] Person, E. S. , E. A. Lacey , and J. E. Smith . 2024. “Space Use and Social Networks in California Ground Squirrels: Correlated but Not Congruent Components of Social Behaviour.” Animal Behaviour 217: 39–51.

[ecy4499-bib-0052] Person, E. S. , K. P. von Maydell , J. E. Baldoza , E. A. Lacey , and J. E. Smith . 2023. “Effects of Sample Collection and Storage Methods on Fecal Bacterial Diversity in California Ground Squirrels (*Otospermophilus beecheyi*).” Journal of Mammalogy 104: 1133–1143.

[ecy4499-bib-0053] Petraitis, P. S. , A. E. Dunham , and P. H. Niewiarowski . 1996. “Inferring Multiple Causality: The Limitations of Path Analysis.” Functional Ecology 10: 421.

[ecy4499-bib-0054] Poran, N. S. , R. G. Coss , and E. Benjamini . 1987. “Resistance of California Ground Squirrels (*Spermophilus beecheyi*) to the Venom of the Northern Pacific Rattlesnake (*Crotalus viridis oreganus*): A Study of Adaptive Variation.” Toxicon 25: 767–777. 3672545 10.1016/0041-0101(87)90127-9

[ecy4499-bib-0055] R Core Team . 2021. R: A Language and Environment for Statistical Computing. Vienna: R Foundation for Statistical Computing.

[ecy4499-bib-0056] Rutz, C. , M. C. Loretto , A. E. Bates , S. C. Davidson , C. M. Duarte , W. Jetz , M. Johnson , et al. 2020. “COVID‐19 Lockdown Allows Researchers to Quantify the Effects of Human Activity on Wildlife.” Nature Ecology & Evolution 4(9): 1156–1159. 32572222 10.1038/s41559-020-1237-z

[ecy4499-bib-0057] Santillán, V. , M. Quitián , B. A. Tinoco , E. Zárate , M. Schleuning , K. Böhning‐Gaese , and E. L. Neuschulz . 2020. “Direct and Indirect Effects of Elevation, Climate and Vegetation Structure on Bird Communities on a Tropical Mountain.” Acta Oecologica 102: 103500.

[ecy4499-bib-0058] Shipley, B. 2009. “Confirmatory Path Analysis in a Generalized Multilevel Context.” Ecology 90: 363–368. 19323220 10.1890/08-1034.1

[ecy4499-bib-0059] Short, M. L. , C. N. Service , J. P. Suraci , K. A. Artelle , K. A. Field , and C. T. Darimont . 2024. “Ecology of Fear Alters Behavior of Grizzly Bears Exposed to Bear‐Viewing Ecotourism.” Ecology 105: e4317. 38687245 10.1002/ecy.4317

[ecy4499-bib-0060] Sih, A. 1987. “Prey Refuges and Predator‐Prey Stability.” Theoretical Population Biology 31: 1–12.

[ecy4499-bib-0061] Sikes, R. S. 2016. “2016 Guidelines of the American Society of Mammalogists for the Use of Wild Mammals in Research and Education.” Journal of Mammalogy 97: 663–688. 29692469 10.1093/jmammal/gyw078 PMC5909806

[ecy4499-bib-0062] Silva‐Rodríguez, E. A. , N. Gálvez , G. J. F. Swan , J. J. Cusack , and D. Moreira‐Arce . 2021. “Urban Wildlife in Times of COVID‐19: What Can We Infer from Novel Carnivore Records in Urban Areas?” Science of the Total Environment 765: 142713. 33077221 10.1016/j.scitotenv.2020.142713 PMC9757141

[ecy4499-bib-0063] Smith, J. A. , E. Donadio , J. N. Pauli , M. J. Sheriff , and A. D. Middleton . 2019. “Integrating Temporal Refugia into Landscapes of Fear: Prey Exploit Predator Downtimes to Forage in Risky Places.” Oecologia 189: 883–890. 30868375 10.1007/s00442-019-04381-5

[ecy4499-bib-0064] Smith, J. A. , J. P. Suraci , M. Clinchy , A. Crawford , D. Roberts , L. Y. Zanette , and C. C. Wilmers . 2017. “Fear of the Human ‘Super Predator’ Reduces Feeding Time in Large Carnivores.” Proceedings of the Royal Society B: Biological Sciences 284(1857): 20170433. 10.1098/rspb.2017.0433 PMC5489723 28637855

[ecy4499-bib-0065] Smith, J. E. , C. Carminito , S. Hamilton , K. L. Newcomb , C. Randt , and S. J. Travenick . 2023. “Sensory Integration of Danger and Safety Cues May Explain the Fear of a Quiet Coyote.” Proceedings of the Royal Society B 290: 20231812. 37876200 10.1098/rspb.2023.1812 PMC10598434

[ecy4499-bib-0066] Smith, J. E. , D. A. Gamboa , J. M. Spencer , S. J. Travenick , C. A. Ortiz , R. D. Hunter , and A. Sih . 2018. “Split between Two Worlds: Automated Sensing Reveals Links between Above‐ and Belowground Social Networks in a Free‐Living Mammal.” Philosophical Transactions of the Royal Society B 373: 20170249. 10.1098/rstb.2017.0249 PMC6030582 29967307

[ecy4499-bib-0067] Smith, J. E. , J. E. Ingbretson , M. M. Miner , E. C. Oestreicher , M. L. Podas , T. A. Ravara , L. M. L. Teles , J. C. Wahl , L. M. Todd , and S. Wild . Forthcoming. “Vole Hunting: Novel Predatory and Carnivorous Behavior by California Ground Squirrels.” Journal of Ethology 1: in revision. 10.1007/s10164-024-00832-6 PMC11717845 39802484

[ecy4499-bib-0068] Smith, J. E. , D. J. Long , I. D. Russell , K. L. Newcomb , and V. D. Muñoz . 2016. “ *Otospermophilus beecheyi* (Rodentia: Sciuridae).” Mammalian Species 48: 91–108.

[ecy4499-bib-0069] Stillfried, M. , P. Gras , K. Börner , F. Göritz , J. Painer , K. Röllig , M. Wenzler , H. Hofer , S. Ortmann , and S. Kramer‐Schadt . 2017. “Secrets of Success in a Landscape of Fear: Urban Wild Boar Adjust Risk Perception and Tolerate Disturbance.” Frontiers in Ecology and Evolution 5: 1–12.

[ecy4499-bib-0070] Suraci, J. P. , M. Clinchy , L. Y. Zanette , and C. C. Wilmers . 2019. “Fear of Humans as Apex Predators Has Landscape‐Scale Impacts from Mountain Lions to Mice.” Ecology Letters 22: 1578–1586. 31313436 10.1111/ele.13344

[ecy4499-bib-0071] Tomich, P. Q. 1962. “The Annual Cycle of the California Ground Squirrel *Citellus beecheyi* .” University of California Publication in Zoology 65: 213–282.

[ecy4499-bib-0072] Toscano, B. J. , N. J. Gownaris , S. M. Heerhartz , and C. J. Monaco . 2016. “Personality, Foraging Behavior and Specialization: Integrating Behavioral and Food Web Ecology at the Individual Level.” Oecologia 182: 55–69. 27170290 10.1007/s00442-016-3648-8

[ecy4499-bib-0073] Wirsing, A. J. , M. R. Heithaus , J. S. Brown , B. P. Kotler , and O. J. Schmitz . 2021. “The Context Dependence of Non‐Consumptive Predator Effects.” Ecology Letters 24: 113–129. 32990363 10.1111/ele.13614

[ecy4499-bib-0074] Zanette, L. Y. , and M. Clinchy . 2019. “Ecology of Fear.” Current Biology 29: R309–R313. 31063718 10.1016/j.cub.2019.02.042

[ecy4499-bib-0075] Zellmer, A. J. , E. M. Wood , T. Surasinghe , B. J. Putman , G. B. Pauly , S. B. Magle , J. S. Lewis , C. A. M. Kay , and M. Fidino . 2020. “What Can We Learn from Wildlife Sightings during the COVID‐19 Global Shutdown?” Ecosphere 11: e03215. 32834907 10.1002/ecs2.3215 PMC7435357

