# Supplementary material for: Human presence shifts the landscape of fear for a free‐living mammal
Source: Ecology. 2025 Jan 12;106(1):e4499. doi: 10.1002/ecy.4499 (PMC11725700; doi:10.1002/ecy.4499)
Supplement: Supplementary file 1 — Appendix S1. [file ECY-106-e4499-s001.pdf]

Appendix S1:

Human presence shifts the landscape of fear for a free-living mammal

Chelsea A. Ortiz-Jimenez, Sophie Z. Conroy, Erin S. Person, Jasper DeCuir, Gabriella E.C Gall, Andrew Sih, Jennifer E. Smith

Ecology

Table S1. List of each path removed from *a priori* SEM and the associated degrees of freedom (df), Fisher's C, AIC, BIC and P-value change. The final model selected is shown in bolded text, chosen based on lowest BIC value.

Round Number	df	Fischer's C	AIC	BIC	P-value	Path removed
1	6	36.136	152.136	365.784	0	
2	8	36.151	150.151	360.115	0	Remove non-target visitor from mean time per visit
3	10	36.244	148.244	354.524	0	Remove sky cover from proportion millet remaining
4	12	36.346	146.346	348.943	0	Remove rattlesnake activity from mean time per visit
5	14	36.481	144.481	343.394	0.001	Remove rattlesnake activity from proportion millet remaining
6	16	36.716	142.716	337.946	0.002	Remove distance from nearest burrow from raptor activity
7	18	37.001	141.001	332.547	0.005	Remove squirrel density from mean time per visit
8	20	37.512	139.512	327.375	0.010	Remove human & dog activity from number of unique foragers
9	22	38.739	138.739	322.918	0.015	Remove distance from nearest burrow from mean time per visit
10	24	39.966	137.966	318.461	0.022	Remove non-target visitor from proportion of millet remaining
11	26	41.829	137.829	314.641	0.026	Remove ground cover from mean time per visit
12	28	44.345	138.345	311.473	0.026	Remove ground cover from proportion millet remaining
13	30	46.211	138.211	307.656	0.030	Remove rattlesnake activity from number of unique foragers
14	20	8.640	98.640	264.401	0.987	Remove non-target visitor from number of unique foragers
15	22	10.683	98.683	260.761	0.979	Remove sky cover from squirrel density
16	24	13.249	99.249	257.643	0.962	Remove raptor from proportion millet remaining
17	26	18.350	102.35	257.060	0.863	Remove human & dog activity from mean time per visit
18	28	21.882	103.882	254.909	0.787	Remove raptor activity from mean time per visit
19	30	27.151	107.151	254.494	0.615	Remove raptor activity from number of unique foragers
20	32	32.784	110.784	254.444	0.428	Remove raptor activity from squirrel density
21	34	44.107	120.107	260.083	0.115	Remove sky cover from mean time per visit
22	32	29.391	107.391	251.051	0.559	Add ground cover to mean time per visit
23	34	37.986	113.986	253.962	0.293	Remove ground cover from squirrel density
**24**	**32**	**28.896**	**106.896**	**250.556**	**0.624**	**Add sky cover to squirrel density**

Table S2. List of each path removed from *a priori* SEM and the associated degrees of freedom (df), Fisher's C, AIC, BIC and P-value change. The final model selected is shown in bolded text, chosen based on lowest BIC value.

Round Number	df	Fischer's C	AIC	BIC	*P*-value	Path removed
1	6	7.921	123.921	342.120	0.244	
2	8	7.972	121.972	336.409	0.436	Remove rattlesnake activity from number of unique foragers
3	10	8.174	120.174	330.849	0.612	Remove squirrel density from mean time per visit
4	12	8.322	118.322	325.235	0.760	Remove raptor activity from mean time per visit
5	14	8.967	116.967	320.118	0.833	Remove rattlesnake activity from proportion millet remaining
6	16	9.663	115.663	315.052	0.884	Remove ground cover from rattlesnake activity
7	18	10.427	114.427	310.054	0.917	Remove non-target visitor from mean time per visit
8	20	11.212	113.212	305.077	0.941	Remove human & dog activity from mean time per visit
9	22	12.261	112.261	300.364	0.952	Remove distance dearest burrow from proportion millet remaining
10	24	13.441	111.441	295.782	0.958	Remove distance from nearest burrow from squirrel density
11	26	15.339	111.339	291.917	0.951	Remove squirrel density from proportion millet remaining
12	28	17.263	111.263	288.079	0.943	Remove sky cover from number of unique foragers
13	30	19.022	111.022	284.076	0.940	Remove raptor activity from rattlesnake activity
14	32	21.259	111.259	280.551	0.926	Remove rattlesnake activity from mean time per visit
15	34	23.574	111.574	277.104	0.910	Remove distance from nearest burrow from mean time per visit
16	36	26.443	112.443	274.211	0.878	Remove non-target visitor from percent millet remaining
17	38	29.352	113.352	271.358	0.842	Remove sky cover from squirrel density
18	40	32.602	114.602	268.846	0.791	Remove sky cover from raptor activity
19	30	21.545	101.545	252.027	0.870	Remove non-target visitor from number of unique foragers
20	32	25.965	103.965	250.685	0.765	Remove sky cover from rattlesnake activity
21	34	29.552	105.552	248.510	0.685	Remove distance from nearest burrow from rattlesnake activity
22	36	34.565	108.565	247.761	0.537	Remove human & dog activity from squirrel density
23	38	39.940	111.940	247.374	0.384	Remove raptor activity from proportion millet remaining
**24**	**40**	**42.608**	**112.608**	**244.280**	**0.360**	**Remove human activity from proportion millet remaining**
25	42	48.384	116.384	244.294	0.231	Remove raptor activity from number of unique foragers

Table S3. Standardized direct, indirect, and net effects of variables on number of unique foragers in a) 2019 and b) 2020. Confidence intervals were calculated using 5,000 bootstrapping resamples. Dashed lines indicate variables were not retained in the final model.

Predictor Variables	2019- Standard estimates (95% confidence intervals)			2020- Standard estimates (95% confidence intervals)		
	Direct	Indirect	Net	Direct	Indirect	Net
Mean time per visit	---	---	---	---	---	---
Squirrel density	**0.357** (0.205,0.442)	---	**0.357** (0.205,0.442)	**0.190** (-0.004,0.254)	---	**0.190** (-0.004,0.254)
Human & dog activity	---	**-0.089** (-0.165,-0.012)	**-0.089** (-0.165,-0.012)	**-0.256** (-0.387,-0.131)	**0.067** (0.030,0.106)	**-0.189** (-0.313,-0.081)
Rattlesnake activity	---	**0.098** (0.040,0.167)	**0.098** (0.040,0.167)	---	**0.036** (-0.004,0.074)	**0.036** (-0.004,0.074)
Raptor activity	---	**0.012** (-0.008,0.042)	**0.012** (-0.008,0.042)	**-0.128** (-0.275,-0.048)	**-0.089** (-0.121,0.002)	**-0.217** (-0.311,-0.121)
Distance to burrow	**-0.148** (-0.260,-0.068)	**-0.140** (-0.191,-0.080)	**-0.288** (-0.382,-0.204)	**-0.280** (-0.386,-0.197)	**-0.026** (-0.073,0.008)	**-0.306** (-0.413,-0.221)
Ground cover	**-0.239** (-0.353,-0.129)	**-0.037** (-0.079,-0.013)	**-0.276** (-0.391,-0.164)	**-0.257** (-0.357,-0.149)	**0.006** (-0.069,0.045)	**-0.251** (-0.365,-0.140)
Sky cover	**-0.140** (-0.234,-0.044)	**0.038** (-0.024,0.094)	**-0.102** (-0.213,0.000)	---	---	---
Non-target forager	---	---	---	---	---	---

Table S4. Standardized direct, indirect, and net effects of variables on the average time a forager spent on a plate (mean time per visit) in a) 2019 and b) 2020. Confidence intervals were calculated using 5,000 bootstrapping resamples. Dashed lines indicate variables were not retained in the final model.

**Predictor Variables**	**2019- Standard estimates** (95% confidence intervals)			**2020- Standard estimates** (95% confidence intervals)		
	**Direct**	**Indirect**	**Net**	**Direct**	**Indirect**	**Net**
Number of unique foragers	**0.747** (0.700,0.818)	---	**0.747** (0.700,0.818)	**0.639** (0.513,0.690)	---	**0.639** (0.513,0.690)
Squirrel density	---	**0.266** (0.166,0.342)	**0.266** (0.166,0.342)	---	**0.122** (-0.003,0.159)	**0.122** (-0.003,0.159)
Human & dog activity	---	**-0.066** (-0.127,-0.014)	**-0.066** (-0.127,-0.014)	---	**-0.121** (-0.204,-0.042)	**-0.121** (-0.204,-0.042)
Rattlesnake activity	---	**0.073** (0.031,0.128)	**0.073** (0.031,0.128)	---	**0.023** (-0.003,0.047)	**0.023** (-0.003,0.047)
Raptor activity	---	**0.009** (-0.005,0.032)	**0.009** (-0.005,0.032)	---	**-0.139** (-0.200,-0.069)	**-0.139** (-0.200,-0.069)
Distance to burrow	---	**-0.216** (-0.296,-0.157)	**-0.216** (-0.296,-0.157)	---	**-0.196** (-0.260,-0.131)	**-0.196** (-0.260,-0.131)
Ground cover	**0.089** (0.033,0.158)	**-0.206** (-0.300,-0.129)	**-0.117** (-0.219,-0.016)	**0.110** (0.040,0.172)	**-0.161** (-0.235,-0.084)	**-0.051** (-0.140,0.037)
Sky cover	---	**-0.077** (-0.162,-0.006)	**-0.077** (-0.162,-0.006)	**0.119** (0.048,0.186)	---	**0.119** (0.048,0.186)
Non-target forager	---	---	---	---	---	---

Table S5. Mean ± standard error for different visitor types in 2019 and 2020 for the entire site.

Visitor	Mean ± S.E.	
	**2019**	**2020**
Human & dog	1.008 ± 0.068	1.144 ± 0.102
Rattlesnake	0.009 ±0.001	0.006 ± 0.001
Raptor	0.014 ±0.001	0.026 ± 0.002

Table S6. Mean ± standard error for different visitor types in 2019 and 2020 within each region across the site.

Year	Region	Activity (Mean ± Standard error)			
		Rattlesnake	*Raptor*	*Human & Dog*	*Squirrel density*
2019	1	0.028 ± 0.01	0.006 ± 0.00	2.867 ± 0.40	0.116 ± 0.04
	2	0.016 ± 0.01	0.006 ± 0.00	1.017 ± 0.21	0.230 ± 0.05
	3	0.004 ± 0.00	0.014 ± 0.00	0.858 ± 0.12	0.289 ± 0.07
	4	0.001 ± 0.00	0.010 ± 0.00	0.493 ± 0.09	0.069 ± 0.02
	5	0.003 ± 0.00	0.016 ± 0.00	0.732 ± 0.15	0.048 ± 0.01
	6	0.000 ± 0.00	0.024 ± 0.00	0.210 ± 0.05	0.009 ± 0.00
	7	0.000 ± 0.00	0.041 ± 0.00	0.115 ± 0.00	0.000 ± 4.97
2020	1	0.015 ± 0.00	0.000 ± 0.00	4.297 ± 0.65	0.207 ± 0.07
	2	0.007 ± 0.00	0.000 ± 0.00	0.608 ± 0.08	0.505 ± 0.08
	3	0.002 ± 0.00	0.005 ± 0.00	0.616 ± 0.07	0.491 ± 0.05
	4	0.005 ± 0.00	0.022 ± 0.00	0.598 ± 0.08	0.172 ± 0.02
	5	0.001 ± 0.00	0.051 ± 0.01	0.332 ± 0.03	0.135 ± 0.02
	6	0.003 ± 0.00	0.083 ± 0.01	0.261 ± 0.03	0.048 ± 0.01
	7	0.004 ± 0.00	0.094 ± 0.00	0.543 ± 0.07	0.003 ± 0.00

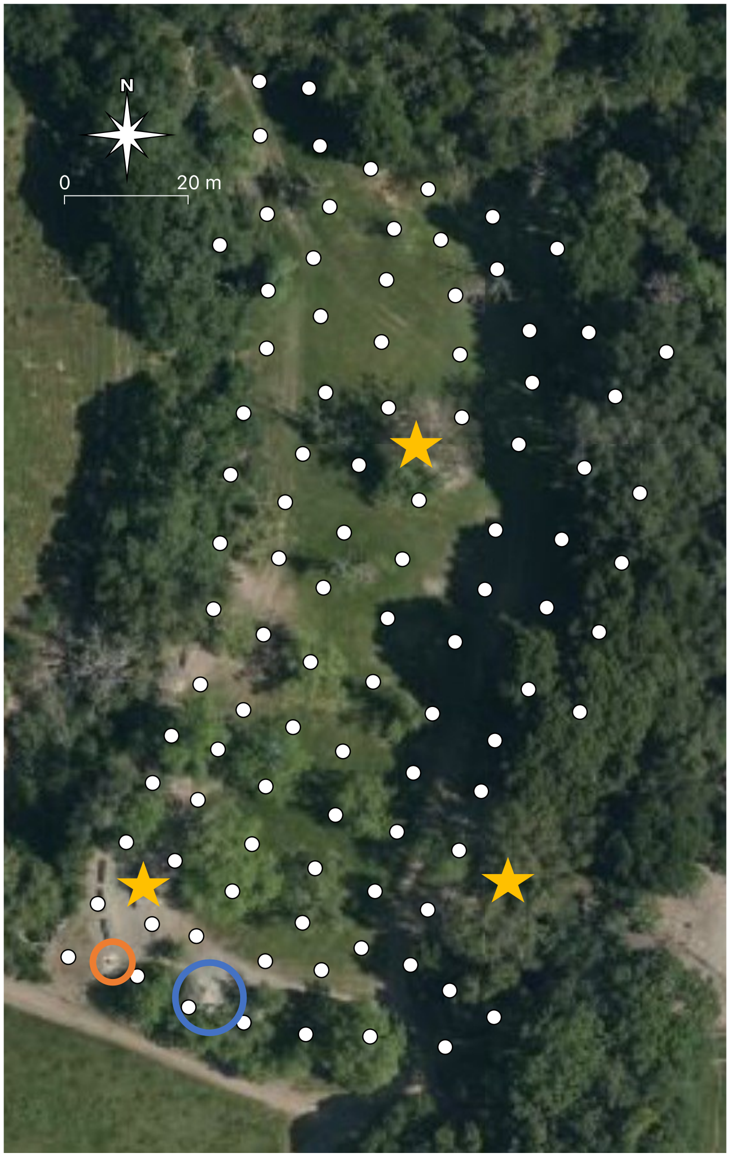

Figure S1. Aerial image of study site at Briones Regional Park, Contra Costa County, California, U.S.A. White dots represent plate locations for one of the two years. Gold stars represent locations where observers quietly sit on picnic benches during experiment to record ground squirrel visitations at each individual plate. Blue circle denotes location of an outhouse and orange circle denotes location of water fountain often used by humans entering the park. Aerial imagery of study site © Google, Map data © 2021.
